# Production of infectious human immunodeficiency virus type 1 does not require depletion of APOBEC3G from virus-producing cells

**DOI:** 10.1186/1742-4690-1-27

**Published:** 2004-09-17

**Authors:** Sandra Kao, Eri Miyagi, Mohammad A Khan, Hiroaki Takeuchi, Sandrine Opi, Ritu Goila-Gaur, Klaus Strebel

**Affiliations:** 1 Laboratory of Molecular Microbiology, Viral Biochemistry Section; National Institute of Allergy and Infectious Diseases, NIH; Building 4, Room 310; 4 Center Drive, MSC 0460; Bethesda, MD 20892-0460, USA

## Abstract

**Background:**

The human immunodeficiency virus Vif protein overcomes the inhibitory activity of the APOBEC3G cytidine deaminase by prohibiting its packaging into virions. Inhibition of APOBEC3G encapsidation is paralleled by a reduction of its intracellular level presumably caused by the Vif-induced proteasome-dependent degradation of APOBEC3G.

**Results:**

In this report we employed confocal microscopy to study the effects of Vif on the expression of APOBEC3G on a single cell level. HeLa cells dually transfected with Vif and APOBEC3G expression vectors revealed efficient co-expression of the two proteins. Under optimal staining conditions approximately 80% of the transfected cells scored double-positive for Vif and APOBEC3G. However, the proportion of double-positive cells observed in identical cultures varied dependent on the fixation protocol and on the choice of antibodies used ranging from as low as 40% to as high as 80% of transfected cells. Importantly, single-positive cells expressing either Vif or APOBEC3G were observed both with wild type Vif and a biologically inactive Vif variant. Thus, the lack of APOBEC3G in some Vif-expressing cells cannot be attributed to Vif-induced degradation of APOBEC3G. These findings are consistent with our results from immunoblot analyses that revealed only moderate effects of Vif on the APOBEC3G steady state levels. Of note, viruses produced under such conditions were fully infectious demonstrating that the Vif protein used in our analyses was both functional and expressed at saturating levels.

**Conclusions:**

Our results suggest that Vif and APOBEC3G can be efficiently co-expressed. Thus, depletion of APOBEC3G from Vif expressing cells as suggested previously is not a universal property of Vif and thus is not imperative for the production of infectious virions.

## Background

Replication of human immunodeficiency virus type 1 (HIV-1) in most primary cells and some immortalized T cell lines is dependent on the expression of a functional Vif protein. In the absence of Vif, virus replication is restricted by a host factor that was recently identified as CEM15 (now referred to as APOBEC3G) [1], a host cytidine deaminase targeting DNA substrates in vitro [2] but whose role in normal cells is unknown. In the absence of Vif, APOBEC3G is efficiently incorporated into virus particles [3-9] where it causes extensive cytidine to uracil changes on the viral minus-strand cDNA during reverse transcription [5,10-12]. The conversion of cytidine to deoxyuridine on the minus-strand cDNA either results in guanine to adenine changes on the viral plus-strand cDNA to yield highly mutated viral genomes or triggers the degradation of the deaminated minus strand cDNA through the action of a DNA repair mechanism that involves removal of the uracil base by uracil DNA glycosylase and subsequent endonucleolytic cleavage at the abasic sites by apyrimidinic endonuclease (reviewed in [13,14]). While both mechanisms are detrimental to virus replication, the reported inability of vif-defective viruses grown in restrictive cells to reverse transcribe the viral genome into full-length cDNA is more consistent with the latter mechanism involving the degradation of deaminated viral cDNA [15-19].

Vif is a 23-kDa basic protein that is expressed late during infection in a Rev-dependent manner [20]. Immunocytochemical analyses revealed a largely cytoplasmic localization of Vif [21-23]. However, Vif is efficiently incorporated into HIV particles during productive infection through an interaction with viral genomic RNA and associates with viral nucleoprotein complexes [22,24-26]. In the presence of Vif, the steady-state levels of cell-associated APOBEC3G – as judged by immunoblot analysis – are reduced by 3–10 fold [3-8,27,28]. This Vif-dependent reduction in APOBEC3G levels has been attributed to proteasome-dependent degradation of the protein and requires a direct interaction of Vif with APOBEC3G [3,6-8].

Like Vif, APOBEC3G is a cytoplasmic protein. In fact, co-immunoprecipitation analyses demonstrated an interaction of Vif and APOBEC3G in transiently transfected cells [3,5,6,27,29-32]. The formation of stable Vif:APOBEC3G complexes seemed to be at odds with the reported proteasome-dependent degradation of APOBEC3G in Vif-expressing cells [3,6-9,27,28]. Indeed, the identification of Vif:APOBEC3G complexes in mixtures of cell extracts that had been individually transfected to express either Vif or APOBEC3G suggested that the stable interaction of Vif and APOBEC3G during co-immunoprecipitation may occur after cell lysis [6]. Thus, the co-immunoprecipitation of Vif and APOBEC3G from cell extracts is not necessarily an indication of the existence of stable intracellular complexes. Quite to the contrary, Marin et al reported a profound effect of Vif on the expression of APOBEC3G on a single cell level. They found that expression of Vif in transiently transfected COS7 cultures resulted in an almost complete segregation of cells expressing either APOBEC3G or Vif [6]. Interestingly, this segregation of Vif and APOBEC3G into separate cells was seen only for wild type Vif. In fact, only 10% of cells expressing wild type Vif were double-positive while 95% of cells expressing an inactive Vif variant also contained APOBEC3G [6].

The current study aims at characterizing in more detail the effects of Vif on the expression of human APOBEC3G on a single cell level. The study was initiated because of the apparent discrepancy between the drastic effects of Vif on APOBEC3G reported by Marin et al and our own finding of only moderate effects of Vif on APOBEC3G expression in transiently transfected cells. In our study, Vif was expressed from a subviral construct in a Tat- and Rev-dependent manner while APOBEC3G was expressed either in a Tat-dependent manner from an HIV-1-LTR-based vector or independently from a CMV-promoter-based expression vector. The Tat-dependent APOBEC3G expression vector was used to restrict APOBEC3G expression to cells also expressing Tat (and thus Vif).

Confocal microscopic analysis of HeLa cells transiently transfected with Vif and APOBEC3G expression vectors revealed significant variations in the number of double-positive cells in identical samples ranging from as low as 40% to as high as 80% of transfected cells depending on fixation method and antibodies employed. Importantly, the appearance of cells expressing only Vif or APOBEC3G was observed both with wild type Vif and a biologically inactive variant and thus cannot be explained by Vif-induced degradation of APOBEC3G. Finally, despite the efficient co-expression of Vif and APOBEC3G, viruses produced in these cultures were fully infectious. We therefore conclude that the Vif-induced exclusion of APOBEC3G from virus-producing cells reported by Marin et al [6] does not apply to our system and because of that is not a universal property of all Vif proteins. This implies that elimination of APOBEC3G is not an obligate requirement for the production of infectious viruses from APOBEC3G-expressing cells.

## Results

### Expression of Vif in the context of a proviral vector only moderately reduces cellular APOBEC3G levels

**Figure 1:**
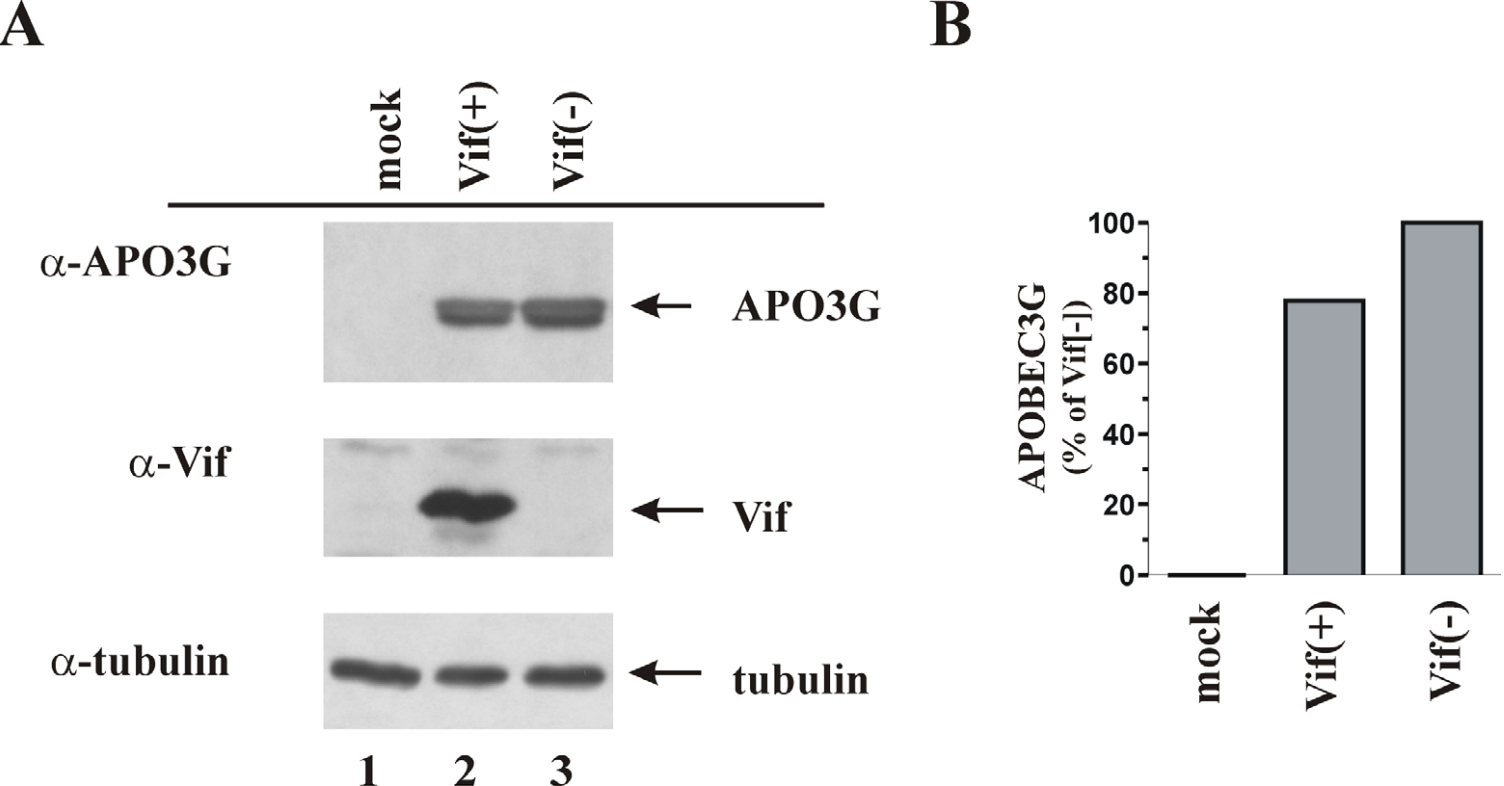
Vif has a moderate effect on APOBEC3G steady-state levels. **(A) **HeLa cells were transfected with pNL-A1 and pcDNA-APO3G vector DNA at a 4:1 ratio. As control, mock-transfected cells (lane 1) and cells transfected with the Vif-deficient pNL-A1ΔVif construct and pcDNA-APO3G vector DNA at a 4:1 ratio (lane 3) were included. Cell lysates were processed for immunoblotting as described in Methods and APOBEC3G and Vif-specific proteins were identified using an APOBEC3G-specific polyclonal antibody (α-APO3G) or a Vif-specific monoclonal antibody #319 (α-Vif). Tubulin was identified using an antibody to α-tubulin. **(B) **APOBEC3G-specific bands were acquired by densitometric scanning of the film and were quantified using the Fuji ImageGauge 4.0 software (Fuji Photofilm Co, LTD). Results are expressed as percent of the Vif-negative control, which was defined as 100%.

A number of previous studies reported the efficient Vif-dependent degradation of APOBEC3G by cellular proteasomes [3,6,8,28]. However, we and others noted only a moderate reduction of the cellular APOBEC3G levels in response to Vif expression [4,5]. This is exemplified in figure 1 where APOBEC3G was expressed either in the presence or absence of Vif. Specifically, HeLa cells were transfected with pcDNA-APO3G together either with wild type pNL-A1 (Fig. 1A, lane 2) or its vif-defective variant, pNL-A1vif(-) (lane 3). Mock transfected cells were included as a control (lane 1). Cells were harvested 24 hr post-transfection and whole-cell lysates were subjected to immunoblot analysis as described in Methods using an APOBEC3G-specific polyclonal antibody (Fig. 1A, top panel) or a Vif-specific monoclonal antibody (Fig. 1A, middle panel). To control for loading errors, the filters were re-probed with an antibody to α-tubulin (Fig. 1A, bottom panel). Consistent with our previous results, expression of Vif from pNL-A1 only moderately reduced the steady-state levels of APOBEC3G in HeLa cells. Quantitation of the data confirmed that expression of APOBEC3G in the presence of Vif was reduced by only about 20% (Fig. 1B).

### Co-expression of APOBEC3G and Vif in HeLa cells

**Figure 2:**
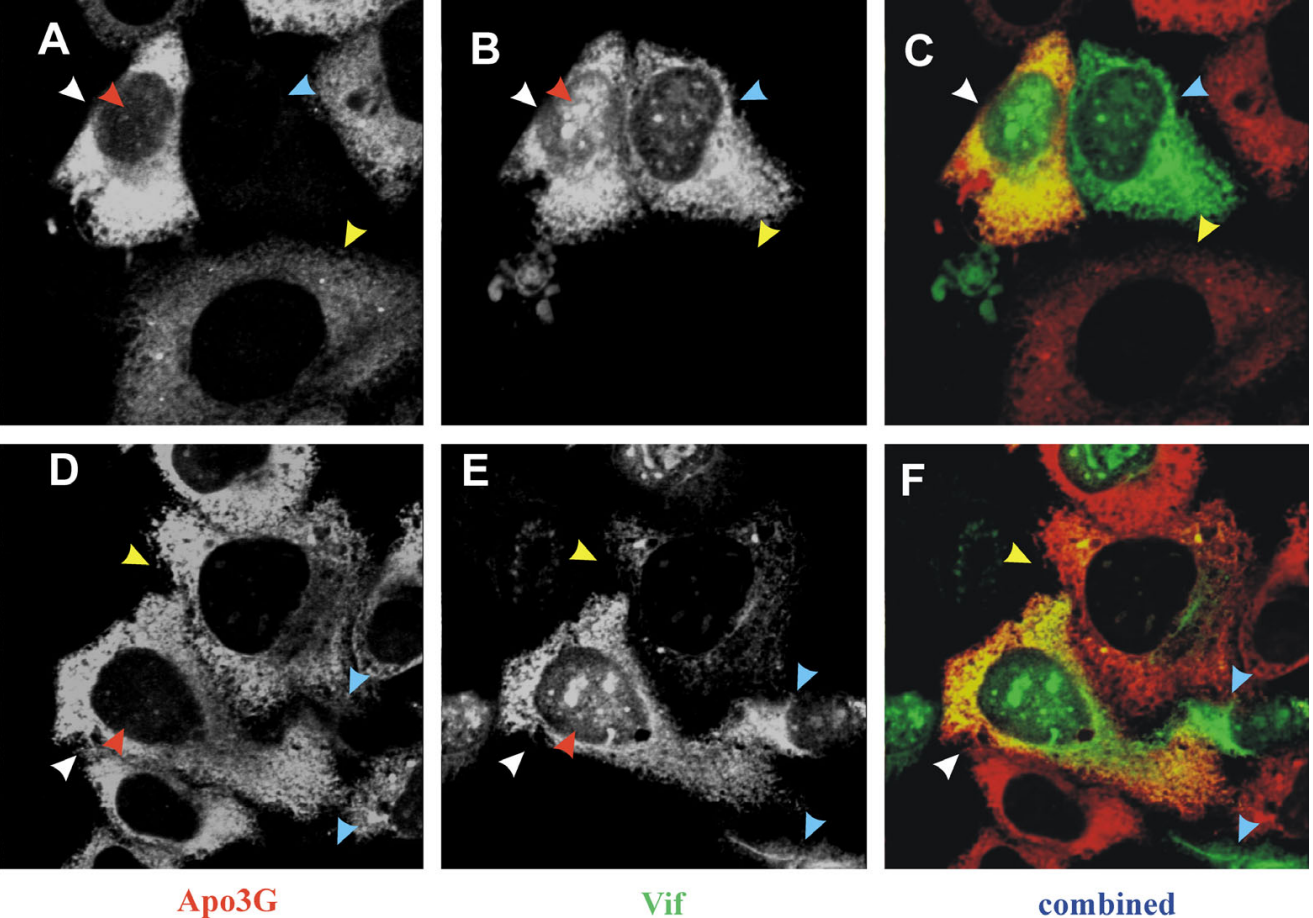
Co-expression of Vif and APOBEC3G in HeLa cells. HeLa cells were transfected with pNL-A1 and pcDNA-APO3G at a 1:1 molar ratio. Transfected cells were grown on cover slips, fixed in methanol and processed for confocal microscopic analysis as described in Methods. Cells were stained with a rabbit polyclonal antibody to APOBEC3G **(A & D) **and a monoclonal Vif antibody **(B & E**). APOBEC3G was visualized using a Texas red-conjugated secondary antibody; Vif was visualized with a Cy2-conjugated secondary antibody. Panels C and F are merged images of panels **A & B **and **D & E**, respectively. Arrow heads are defined as follows: white = APOBEC3G:Vif-double-positive cells; yellow = Vif-negative cells; red = cells exhibiting nuclear and cytoplasmic staining for Vif.

An earlier study investigating the coexpression of Vif and APOBEC3G by confocal microscopy concluded that APOBEC3G was virtually excluded from Vif-expressing COS7 cells [6]. To verify this observation, we investigated the effects of Vif on APOBEC3G expression on a single cell level by performing a series of immunocytochemical analyses. For that purpose, HeLa cells were transfected with the Vif expression vector pNL-A1 together with pcDNA-APO3G for the expression of human APOBEC3G. Cells were grown on cover slips, fixed 24 hr later with cold methanol, and stained with antibodies to APOBEC3G (Fig. 2, panels A & D) and Vif (panels B & E). The results of this experiment show that APOBEC3G can be expressed in Vif-positive cells (white arrow heads) without a dramatic reduction in its expression level when compared to Vif-negative cells (yellow arrow heads). Furthermore, these data confirm that APOBEC3G is localized to the cytoplasm while Vif was observed in this experiment in some cells both in the cytoplasm and the nuclei of cells (red arrow heads). Finally, we also observed cells expressing Vif that were APOBEC3G-negative (blue arrow heads). Overlay of the Vif and APOBEC3G channels revealed a partial co-localization of Vif and APOBEC3G in the cytoplasm apparent by the yellow staining in panels C & F of figure 2. Interestingly, a significant number of cells in this experiment were single-positive expressing either APOBEC3G or Vif alone. The appearance of Vif-positive, APOBEC3G-negative cells could be explained by a Vif-dependent restriction of APOBEC3G expression as proposed by Marin et al [6]. However, cells expressing Vif only were rare when compared to cells expressing APOBEC3G alone (data not shown). The preponderance of APOBEC3G single positive cells cannot be explained by a Vif-dependent restriction but more likely represents a technical, albeit reproducible, artifact.

### Tat-dependent expression of APOBEC3G reduces the fraction of single-positive cells

In the experiment shown in figure 2, APOBEC3G was expressed under the control of a CMV promoter while Vif was expressed from the HIV-LTR promoter under the regulatory control of Tat and Rev. Because of the independent expression of Vif and APOBEC3G it cannot be ruled out that the large number of single-positive cells in that experiment – while statistically improbable – was caused by the selective transfection of cells with either the Vif or the APOBEC3G expression vector. To check this possibility, we expressed APOBEC3G from the HIV-1 long terminal repeat (LTR) promoter driven vector pHIV-APO3G [4]. Because of its dependence on Tat, APOBEC3G expression from pHIV-APO3G is restricted to cells also expressing Vif. Indeed, transfection of pHIV-APO3G into cells in the absence of pNL-A1 or any other Tat expression vector did not reveal any APOBEC3G expression as judged by immunofluorescence analysis or immunoblotting attesting to the strict Tat-dependence of this APOBEC3G expression vector (data not shown).

In addition of measuring the context-dependent expression of APOBEC3G, we also wanted to determine the influence of the fixation procedure on the efficiency of Vif:APOBEC3G co-staining. It is well known that the choice of fixative can affect the ability of a given antibody to recognize a specific epitope on its target protein. Frequently, epitopes are masked because of the folding properties of a protein in vivo or because of pre-existing protein-protein interactions that may compete for antibody binding. To test this possibility we compared a formaldehyde fixation procedure employed previously [6] with the methanol fixation procedure employed in our own studies [22].

**Figure 3:**
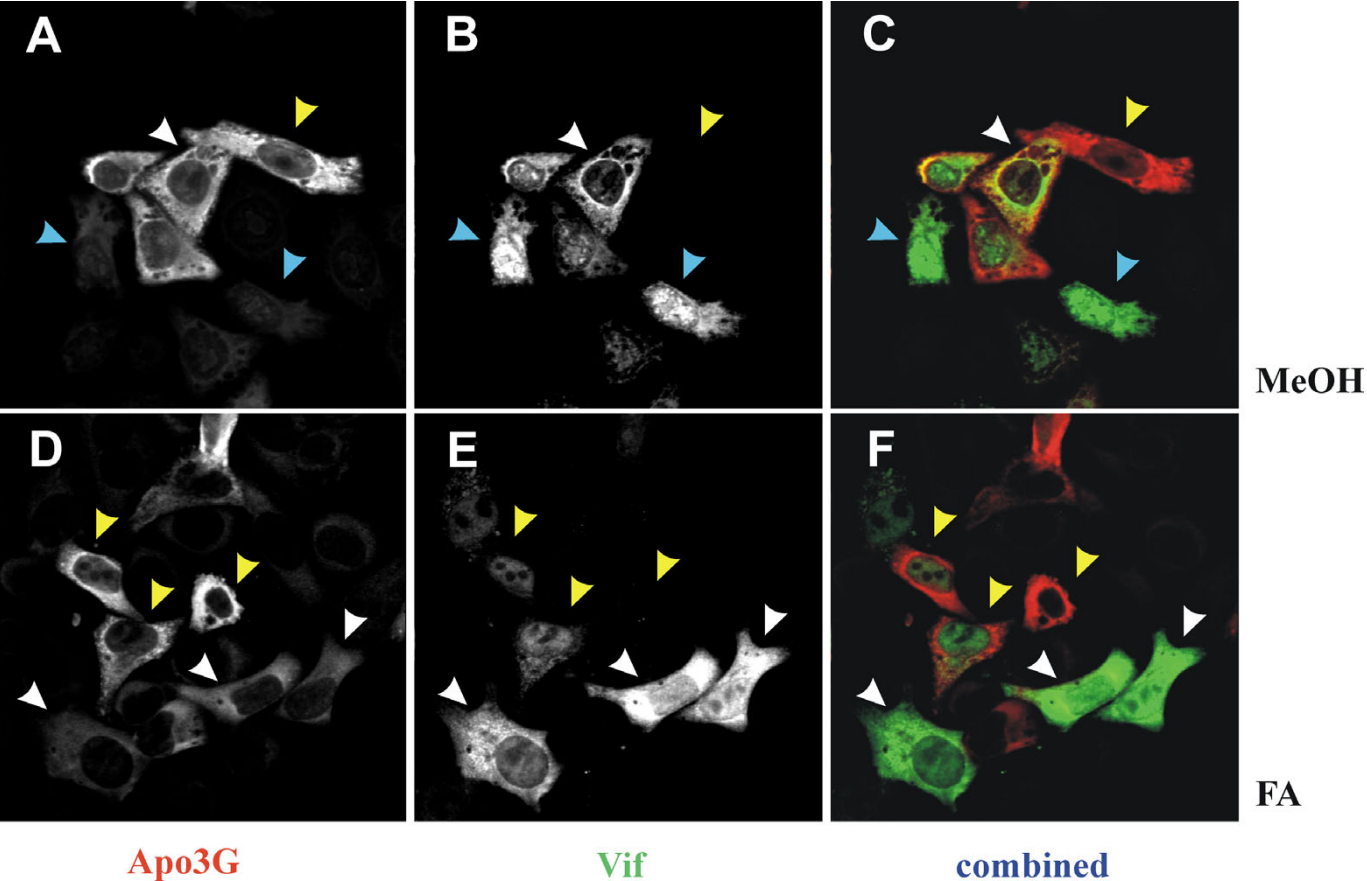
Tat-dependent expression of APOBEC3G. HeLa cells were transfected with pNL-A1 and pHIV-APO3G at a 1:1 molar ratio. Transfected cells were grown on cover slips for 24 hr and then either fixed with ice-cold methanol (panels A-C) or with formaldehyde buffer as described in Methods (panels D-F). Cells were stained with an APOBEC3G-specific antibody (A & D) and a Vif monoclonal antibody (B & E) as in figure 2 and analyzed on a confocal microscope. Panels C & F are overlays of panels A & B and D & E, respectively. Arrow heads are defined as follows: white = APOBEC3G:Vif-double-positive cells; yellow = Vif-negative cells; blue = APOBEC3G negative cells.

HeLa cells were transfected with pNL-A1 and pHIV-APO3G at a 1:1 molar ratio. Cells were fixed 24 hr later either with methanol (MeOH) as in figure 2 or with formaldehyde (FA) as described in Methods. Cells were stained with Vif- and APOBEC3G-specific antibodies as described in figure 2. The results of this experiment show that expression of APOBEC3G under the control of the HIV-1 LTR indeed increased the proportion of double-positive cells both in methanol-fixed samples (Fig. 3, panels A-C) and formaldehyde-fixed specimens (Fig. 3, panels D-F). This suggests that the high proportion of single-positive cells observed in figure 2 was not the result of a Vif-dependent restriction of APOBEC3G but was caused by the independent expression of APOBEC3G from a Tat-independent promoter. Again, in methanol-fixed samples APOBEC3G expression levels in Vif-positive cells (Fig. 3A, white arrow heads) were indistinguishable from those observed for neighboring Vif-negative cells (Fig. 3A, yellow arrow head). Interestingly, the APOBEC3G fluorescent intensity appeared to be reduced in Vif-positive formaldehyde-fixed specimens when compared to Vif-negative cells or cells expressing low levels of Vif (Fig. 3D; compare white and yellow arrow heads). Because the methanol-fixed samples did not show a Vif-dependent reduction in APOBEC3G signals in these experiments, we conclude that the reduction in APOBEC3G signals observed in formaldehyde-fixed samples is not the result of Vif-induced degradation of APOBEC3G but is a technical artifact.

### Co-expression of Vif and APOBEC3G: Protein degradation or epitope masking?

**Figure 4:**
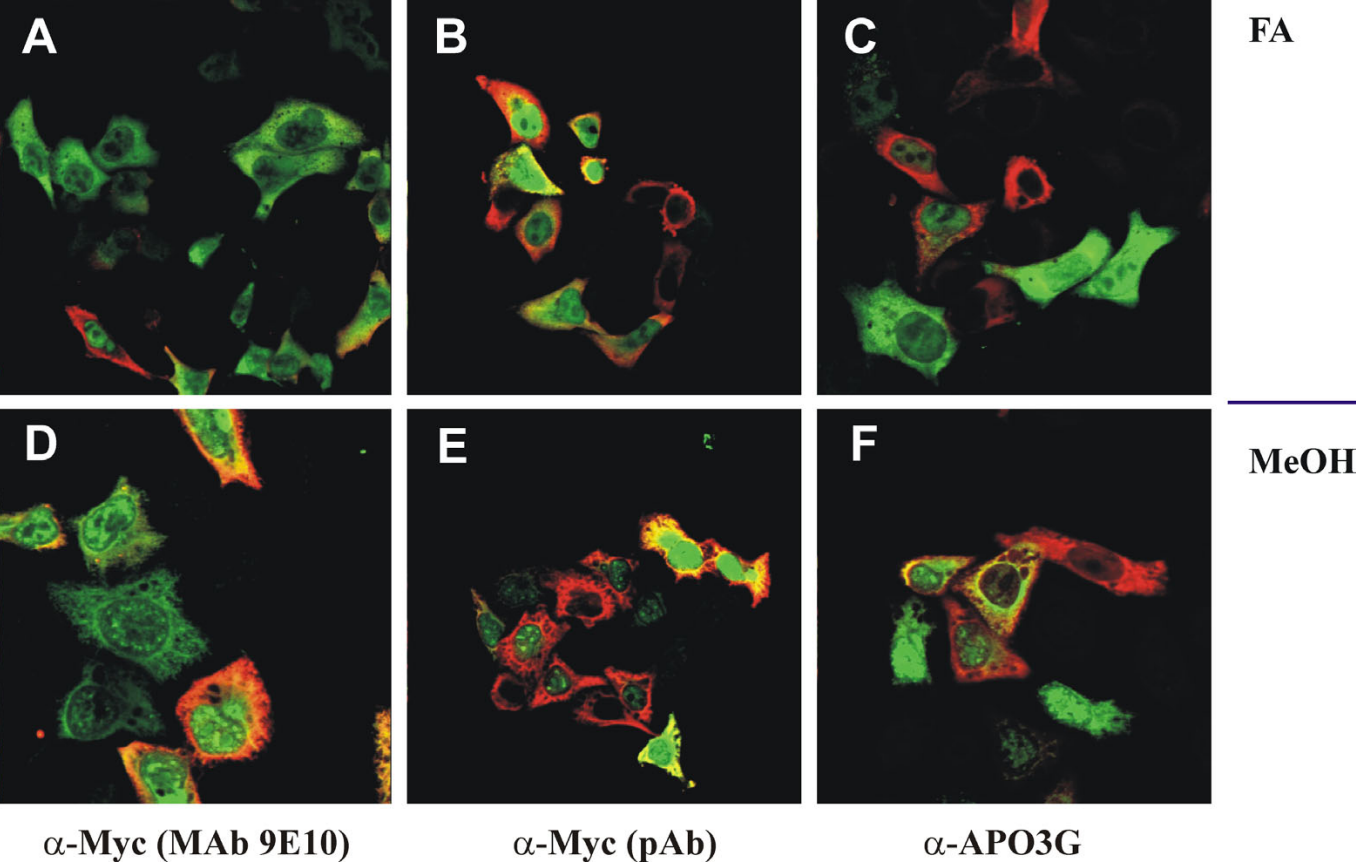
Effect of fixation method and antibody choice on co-expression of Vif and APOBEC3G. HeLa cells were transfected with pNL-A1 and pHIV-APO3G as described in figure 3. Cells were grown on cover slips for 24 hr and then either fixed with ice-cold methanol (panels A-C) or with formaldehyde buffer as in figure 3 (panels D-F) and stained with the following combinations of antibodies: (A & D) polyclonal Vif + anti-Myc MAb 9E10; (B & E) anti-Vif MAb #319 + anti-Myc polyclonal antibody; (C & F) anti-Vif MAb #319 + anti-APO3G polyclonal antibody. Vif was visualized using Cy2-conjugated secondary antibodies (green) and APOBEC3G was visualized with Texas red-conjugated antibodies (red). Areas of overlap appear as yellow.

For a more quantitative analysis and to determine possible effects that arise from the use of different antibodies, we extended the experiment shown in figure 3 to include three different antibodies for the identification of APOBEC3G. As before, HeLa cells were co-transfected with a 1:1 ratio of pNL-A1 and pHIV-APO3G plasmid DNAs. Cells were grown on cover slips and fixed 24 hr later either with formaldehyde (Fig. 4, panels A-C) or methanol (panels D-F) as described in figure 3. Cells were then stained with either a monoclonal antibody to the C-terminal Myc-epitope in APOBEC3G together with a polyclonal Vif antibody (Fig. 4, panels A & D), or a polyclonal Myc antibody together with a monoclonal Vif antiserum (Fig. 4, panels B & E). A third set of cells was stained with a polyclonal APOBEC3G-specific antiserum together with the monoclonal Vif antibody (Fig. 4, panels C & F). Representative fields are shown for each combination.

**Figure 5:**
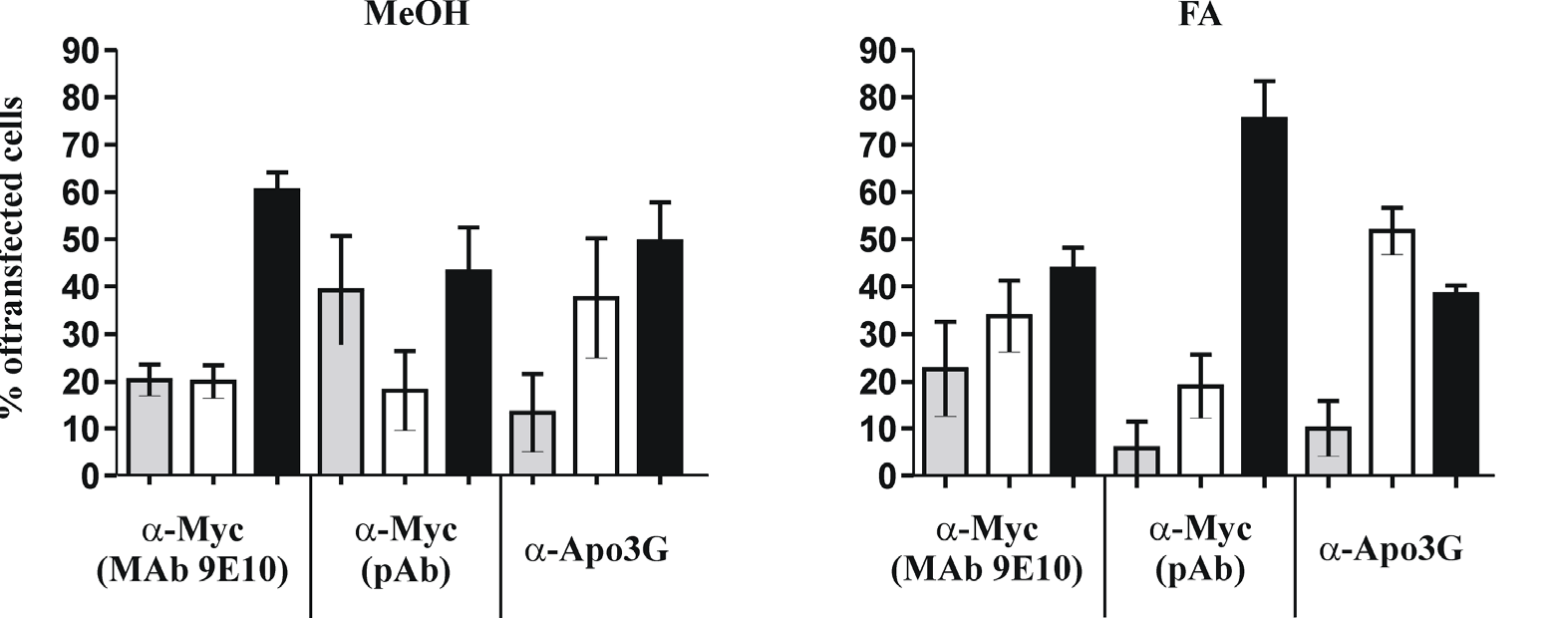
Quantitative analysis of Vif and APOBEC3G co-expression. Samples from figure 4 were analyzed for the expression of Vif (grey bars) or APOBEC3G (white bars) or for double-positive cells (black bars). Between 5 and 10 independent optical fields were analyzed to yield at least 100 transfected cells. Error bars reflect standard deviations calculated from multiple optical fields. The results obtained with methanol-fixed samples (MeOH) are on the left; results from formaldehyde-fixed samples (FA) are on the right.

To quantify the results, multiple optical fields were analyzed (n = 5–10) with a total of at least 100 transfected cells for each parameter. As can be seen in figure 5, methanol-fixed samples showed a relatively modest variation among the three antibodies used. All three antibodies identified between 45% to 60% of the cells as double-positive for Vif and APOBEC3G. In contrast, formaldehyde-fixed samples exhibited a larger antibody-dependent variation. Staining with the 9E10 monoclonal antibody to the Myc-epitope in APOBEC3G yielded the lowest efficiency of staining and identified little more than 40% of the transfected cells as double-positive for Vif and APOBEC3G. In contrast, staining of APOBEC3G was more efficient with the polyclonal Myc antibody, which identified approximately 80% of the transfected cells as double-positive in formaldehyde-fixed samples. Finally, the polyclonal APOBEC3G-specific antibody was slightly less efficient for the staining of FA-fixed samples than methanol-fixed samples and identified about 40% of the formaldehyde-fixed samples as double-positive. Since all samples were derived from the same transfected culture, variations in the co-expression of Vif and APOBEC3G in the individual samples can only be explained by the differential sensitivity of the antibodies to the fixation procedure.

### Exclusion of APOBEC3G from cells expressing biologically inactive Vif protein

**Figure 6:**
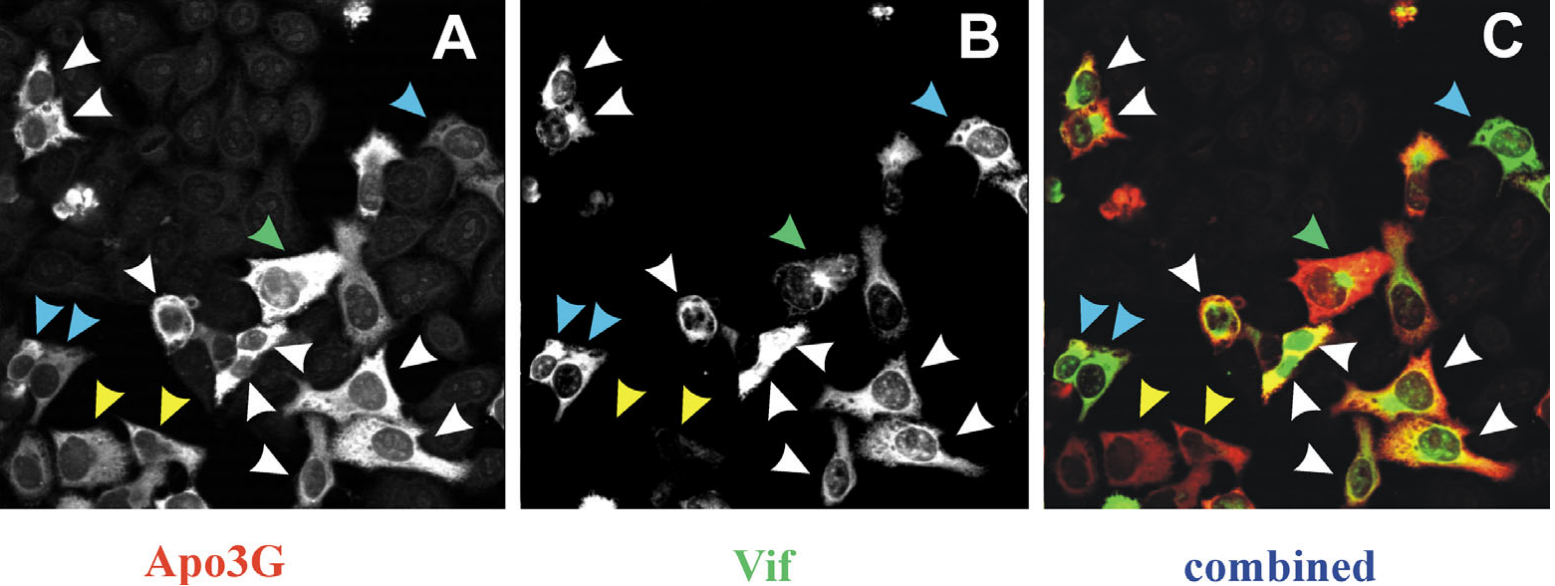
Co-expression of APOBEC3G and a biologically inactive Vif variant. HeLa cells were transfected with pHIV-APO3G and pNL-A1/VifΔ23–43, encoding a biologically inactive Vif variant. Cells were fixed in methanol and stained with the monoclonal Vif antibody (MAb #319; green) and a rabbit polyclonal antibody to APOBEC3G (red) as described above. APOBEC3G is shown in panel A; panel B depicts samples stained for Vif; panel C is the merged image of panels A & B. White and yellow arrow heads depict APOBEC3G:Vif double-positive and Vif-negative cells, respectively. Blue arrow heads point to double-positive cells that show reduced levels of APOBEC3G; the green arrow head depicts a cell where Vif is concentrated around the microtubule organizing center without a similar effect on APOBEC3G.

Under optimal conditions, wild type Vif and APOBEC3G were coexpressed in about 80% of transfected cells (see figure 5). Thus, 20% of the transfected cell population either was expressing Vif but not APOBEC3G or was single-positive for APOBEC3G. To investigate whether the presence of such single-positive cells is due to an activity of Vif or is a general characteristic of transiently transfected cells, we studied the effects of a biologically inactive Vif variant. For this purpose, we employed a Vif mutant carrying a deletion of residues 23–45 in Vif. We previously showed that this mutant is unable to rescue viral infectivity in APOBEC3G-expressing cells [4]. Like wild type Vif, VifΔ23–43 was expressed in the context of the subviral expression vector pNL-A1. HeLa cells were cotransfected with pNL-A1/VifΔ23–43 and pHIV-APO3G, fixed with methanol and processed for confocal microscopy as described for figure 2. As shown in figure 6, coexpression of VifΔ23–43 and APOBEC3G yielded a significant number of double-positive cells (white arrow heads). However, as observed before with wild type Vif, we also identified cells that were Vif-positive but had significantly reduced APOBEC3G levels (Fig. 6, blue arrow heads) or cells that were APOBEC3G positive but did not express Vif (yellow arrow heads). As with wild type Vif, overlay of the Vif and APOBEC3G image channels suggested a partial colocalization of the two proteins. In cells, in which Vif had spontaneously collapsed into a perinuclear aggregate (green arrow head), APOBEC3G did not exhibit a similar change in subcellular distribution. This is in contrast to the Vif-induced reorganization of vimentin reported previously [22]. Thus, VifΔ23–43 is either unable to interact with APOBEC3G or forms complexes that are unstable. These results also imply that the partial colocalization of APOBEC3G and Vif noted in this study may not reflect a true physical interaction of the two proteins.

### Rescue of viral infectivity and Vif-induced reduction of cellular APOBEC3G levels are not directly linked

**Figure 7:**
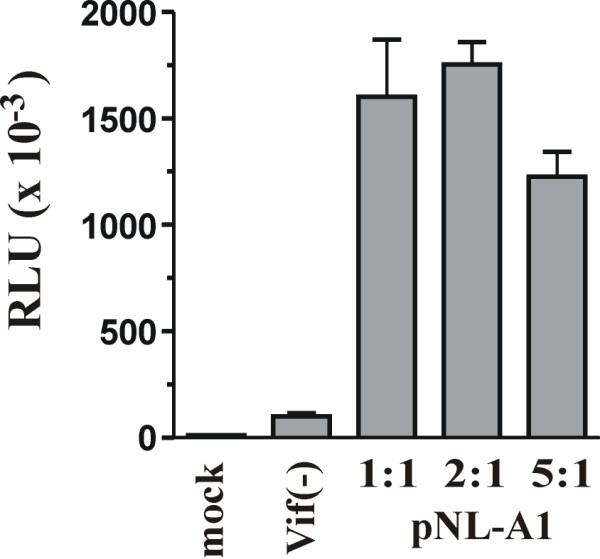
Vif efficiently rescues viral infectivity. HeLa cells were transfected with the vif-defective proviral vector pNL4-3vif(-) together with pNL-A1 and pHIV-APO3G at 1:1, 2:1, or 5:1 molar ratios. Cell lysates and purified, concentrated viral extracts were analyzed by immunoblotting using antibodies to APOBEC3G (APO3G), Vif (MAb #319), or an HIV-positive human serum for the identification of viral capsid protein (CA). Virus-containing, filtered supernatants were normalized for equal reverse transcriptase activity and used for the infection of the LuSIV indicator cell line [38]. Virus-induced luciferase activity was measured 24 hr after infection as described in Methods. Relative light units (RLU), which are directly proportional to the infectivity of the viruses, are shown. Error bars reflect standard deviations from duplicate experiments.

The combined results from the experiments shown in figures 2,3,4,5,6, do not support the notion that Vif expression leads to the elimination of APOBEC3G from Vif-positive cells. It can be argued, however, that under the experimental conditions employed in our experiments, the Vif expression levels were insufficient or ineffective. To control for this possibility, we compared the infectivity of viruses produced in the presence of various ratios of Vif and APOBEC3G. To allow a direct comparison with the experiments shown in figures 2 to 6, Vif and APOBEC3G were expressed in trans from pNL-A1 and pHIV-APO3G respectively in the presence of a Vif-defective NL4-3 proviral vector. The ratios of Vif to APOBEC3G expression vector were 1:1, 2:1, and 5:1, respectively. Note that the Vif to APOBEC3G ratio in the experiments shown in figures 2 to 4 was 1:1 throughout. A Vif-negative sample was analyzed as negative control. Virus-containing supernatants were harvested 24 hr after transfection, normalized for equal reverse transcriptase activity and used for the infection of LuSIV indicator cells. Relative virus infectivity was determined by comparing the Tat-dependent expression of luciferase in the target cells (Fig. 7). Interestingly, viruses produced at the lowest Vif:APOBEC3G ratio were virtually as infectious as viruses produced in the presence of higher levels of Vif. In fact, increasing the Vif:APOBEC3G ratio to 2:1 or 5:1 did not significantly increase viral infectivity. Instead, at the 5:1 ratio viral infectivity was slightly reduced, presumably due to the inhibitory effect of Vif at high concentrations as reported previously [39]. Taken together, our data suggest that the inability of Vif to prevent co-expression of APOBEC3G in transiently transfected HeLa cells is not caused by sub-optimal levels of Vif or a lack of Vif activity in our system.

## Discussion

APOBEC3G is able to deaminate cytidine residues on the HIV minus-strand cDNA and cause hypermutation of the viral genome. Nevertheless, HIV-1 is able to efficiently replicate in APOBEC3G expressing cells thanks to the activity of the accessory protein Vif. One of the prerequisites for the antiviral activity of APOBEC3G is that it is packaged into the virions where it selectively targets the viral minus-strand cDNA [5,10-12,40,41] and there is convincing evidence that HIV-1 Vif plays an important role in inhibiting the encapsidation of APOBEC3G. The question of how Vif accomplishes this remains under investigation. A number of groups have reported on the rapid Vif-induced degradation of APOBEC3G by cellular proteasomes [3,6-9,27,28]. Consistent with this, treatment of cells with proteasome inhibitors was found to increase APOBEC3G expression levels despite the presence of Vif [6,7,9,28]. This is contrasted by other reports that noted only a moderate effect of Vif on cellular APOBEC3G levels [4,5]. In fact, our own studies with proteasome inhibitors did not yield a significant increase in APOBEC3G levels in the presence of Vif (manuscript in preparation). Nevertheless, the currently prevailing opinion is that Vif inhibits the encapsidation of APOBEC3G by inducing its rapid degradation in virus-producing cells.

While the results from our own study argue against a depletion of APOBEC3G in Vif-expressing cells – thus implying that Vif can rescue viral infectivity despite the presence of APOBEC3G in virus-producing cells – it is important to point out that our data do not rule out the possibility that Vif – under different experimental conditions – can indeed mediate the proteasome dependent degradation of APOBEC3G. In fact, expression of Vif from a codon-optimized vector consistently had a more pronounced effect on APOBEC3G steady-state levels than Vif expressed from pNL-A1 even though the Vif expression levels from the codon-optimized construct were consistently several-fold lower than those from pNL-A1 (manuscript in preparation). Experiments are ongoing to study the differential effects of Vif expressed from pNL-A1 and Vif expressed from a codon-optimized vector on APOBEC3G stability. However, these results could suggest that the effect of Vif on APOBEC3G steady-state levels may be influenced by the context in which Vif is expressed. At any rate, despite our inability to observe Vif-dependent cellular depletion of APOBEC3G, we were invariably able to recover fully infectious HIV under conditions were the intracellular levels of APOBEC3G were only moderately affected. We therefore conclude that (i) Vif has the ability to rescue viral infectivity even in the presence of APOBEC3G and (ii) that intracellular depletion of APOBEC3G and rescue of viral infectivity may be functionally separable activities of Vif.

For now, the reason for the differences in the sensitivity of APOBEC3G to Vif noted by us versus other research groups remains unexplained. APOBEC3G can form oligomeric structures and is able to interact with Vif. It is therefore possible that such complexes undergo conformational changes that can mask epitopes thus limiting the access of antibodies used in the experiments. Thus, the discrepancy between our findings of the coexpression of Vif and APOBEC3G in the majority of cells and the virtual exclusion of APOBEC3G from Vif-expressing cells reported by Marin et al. [6] may be attributed at least in part to differences in the experimental protocols. It is unlikely that the observed discrepancies are strain-specific variations. To this end we have compared the activities of two HIV-1 Vif isolates, HXB2 and NL4-3, which differ by 18 amino acids (9.4%), and found them to be equally active against APOBEC3G (manuscript in preparation). It is unclear why cotransfection of pHIV-APO3G with pNL-A1 produces a fraction of cells that are single-positive for Vif or for APOBEC3G. Since APOBEC3G expression from the pHIV-APO3G vector is strictly Tat-dependent, the results cannot be explained by differential transfection of cells with individual plasmids. Also, this phenomenon is clearly not a consequence of Vif function, since similar results were observed in the presence of a biologically inactive Vif variant (Fig. 6) or when APOBEC3G was co-expressed with HIV-1 Gag in the absence of Vif (data not shown).

The inability of Vif expressed from pNL-A1 to deplete APOBEC3G is consistent with our previous inability to observe APOBEC3G degradation in kinetic studies [4]. More recent in-depth kinetic analyses of APOBEC3G employing multiple epitope tags and various antibodies confirm these initial findings and suggest that – instead of inducing APOBEC3G degradation – Vif induces conformational changes in APOBEC3G that affect the ability of antibodies to interact with the protein (manuscript in preparation). Experiments are ongoing to study the nature of the APOBEC3G/Vif complexes and to further decipher the mechanism(s) by which Vif inhibits the encapsidation of APOBEC3G under conditions of no or low intracellular degradation.

## Conclusions

Expression of Vif and APOBEC3G in our experimental setup does not lead to the elimination of APOBEC3G from Vif expressing cells. In fact, more than 80% of successfully transfected cells efficiently co-expressed both proteins. Similar results were observed when a biologically inactive Vif variant was co-expressed with APOBEC3G suggesting that the absence of APOBEC3G in some of the Vif-positive cells is not due to Vif-mediated APOBEC3G degradation but reflects a general characteristic of the transient expression system. Moreover, APOBEC3G expression levels were very similar for Vif-positive and Vif-negative cells as judged from the immunostaining consistent with the only modest reduction in APOBEC3G steady-state levels observed in our immunoblot analyses. Nevertheless, viruses produced under such conditions were fully infectious in the presence but not in the absence of Vif attesting to the biological activity of all the proteins involved and demonstrating that Vif was expressed at saturating levels. We conclude that production of infectious viruses from APOBEC3G expressing cells is dependent on Vif but does not necessitate APOBEC3G exclusion from virus-producing cells.

## Methods

### Plasmids

The full-length molecular clone pNL4-3 [33] was used for the production of wild type infectious virus. For transient expression of Vif, the subgenomic expression vector pNL-A1 [34] was employed. This plasmid expresses all HIV-1 proteins except for *gag *and *pol *products. A vif-defective variant of pNL-A1, pNL-A1vif(-) was constructed by deletion of an NdeI/PflMI fragment [4]. Plasmid pNL-A1/VifΔ23–43 expresses a Vif variant carrying a 21 amino acid deletion (residues 23 to 43) in its vif gene as reported elsewhere [4]. This Vif variant is inactive and does not target APOBEC3G [4]. Plasmids pcDNA-APO3G and pHIV-APO3G are vectors for the expression of human APOBEC3G under the control of the CMV immediate early promoter or the HIV promoter, respectively, and were constructed as described elsewhere [4].

### Antisera

Serum from an HIV-positive patient (APS) was used to detect HIV-1-specific capsid (CA) proteins. A monoclonal antibody to Vif (MAb #319) was used for all immunoblot analyses and some of the immunohistochemical analyses as indicated in the text and was obtained from Michael Malim through the NIH AIDS Research and Reference Reagent Program [23,35,37] For all other immunocytochemical analyses our Vif-specific polyclonal antibody (Vif93) was employed. APOBEC3G, carrying a C-terminal Myc epitope tag was identified either with the Myc-specific 9E10 monoclonal antibody or a polyclonal antibodies to the Myc epitope tag (both antibodies were obtained from Sigma-Aldrich, St. Louis). Alternatively, APOBEC3G was identified using a polyclonal rabbit serum against recombinant APOBEC3G [4]. Tubulin was identified using a monoclonal antibody to α-tubulin (Sigma-Aldrich, St. Louis).

### Tissue culture and transfections

HeLa cells were propagated in Dulbecco's modified Eagles medium (DMEM) containing 10% fetal bovine serum (FBS). LuSIV cells are derived from CEMx174 cells and contain a luciferase indicator gene under the control of the SIVmac239 LTR [38]. These cells were obtained through the NIH AIDS Research and Reference Reagent Program and were maintained in complete RPMI 1640 medium supplemented with 10% FBS and hygromycin B (300 μg/ml).

For transfection of HeLa cells, cells were grown in 25 cm^2 ^flasks to about 80% confluency. Cells were transfected using LipofectAMINE PLUS™ (Invitrogen Corp, Carlsbad CA) following the manufacturer's recommendations. A total of 5–6 μg of plasmid DNA per 25 cm^2 ^flask was used. Cells were harvested 24 hr post-transfection. Transfection efficiency in our analyses was generally 30% to 40%.

### Preparation of virus stocks

Virus stocks were prepared by transfecting HeLa cells with appropriate plasmid DNAs. Virus-containing supernatants were harvested 24 hr after transfection. Cellular debris was removed by centrifugation (3 min, 3000 × g) and clarified supernatants were filtered (0.45 μm) to remove residual cellular contaminants. For determination of viral infectivity, unconcentrated filtered viral supernatants were used for the infection of indicator cells. For immunoblot analysis of viral proteins, virus particles (7 ml) were concentrated by ultracentrifugation through 4 ml of 20% sucrose in PBS as described before [4].

### Immunoblotting

For immunoblot analysis of intracellular proteins, whole cell lysates were prepared as follows: Cells were washed once with PBS, suspended in PBS (400 μl/10^7 ^cells), and mixed with an equal volume of sample buffer (4% sodium dodecyl sulfate, 125 mM Tris-HCl, pH 6.8, 10% 2-mercaptoethanol, 10% glycerol, and 0.002% bromphenol blue). Proteins were solubilized by boiling for 10 to 15 min at 95°C with occasional vortexing of the samples to shear chromosomal DNA. Residual insoluble material was removed by centrifugation (2 min, 15000 rpm in Eppendorf Minifuge). Viral proteins were obtained by boiling concentrated viral pellets in a 1:1 mixture of PBS and sample buffer. Cell lysates and viral extracts were subjected to SDS-PAGE; proteins were transferred to PVDF membranes and reacted with appropriate antibodies as described in the text. Membranes were then incubated with horseradish peroxidase-conjugated secondary antibodies (Amersham Biosciences, Piscataway NJ) and visualized by enhanced chemiluminescence (ECL, Amersham Biosciences).

### Infectivity assay

To determine viral infectivity, virus stocks were normalized for equal reverse transcriptase activity and used to infect LuSIV cells (5 × 10^5^) in a 24-well plate total volume 1.2 to 1.4 ml. Cells were incubated for 24 hours at 37°C. Cells were then harvested and lysed in 150 μl of Promega 1x reporter lysis buffer (Promega Corp., Madison WI). To determine the luciferase activity in the lysates, 50 μl of each lysate were combined with luciferase substrate (Promega Corp., Madison WI) by automatic injection and light emission was measured for 10 seconds at room temperature in a luminometer (Optocomp II, MGM Instruments, Hamden CT).

### Immunocytochemistry

For the analysis of transfected HeLa cells, cells were scraped off the flasks 3 hr after transfection and reseeded into 12 well plates containing 0.13 mm cover slips. Cells were grown for 15 to 24 hrs at 37°C in DMEM containing 10% FBS. Cells were then fixed at -20°C in precooled methanol (-20°C) for 10 min followed by two washes in PBS or fixed in FA buffer (5% formaldehyde + 2% sucrose in PBS) for 20 min at room temperature followed by two washes in PBS. Coverslips were stored in PBS at 4°C until use. FA-fixed samples were permeabilized for 30 min at room temperature in permeabilization buffer (1% Triton X-100, 10% sucrose in PBS) prior to incubation with antibodies. For antibody staining, cover slips were incubated in a humid chamber at 37°C for 30 min with primary antibodies at appropriate dilutions in 1% BSA in PBS. Cover slips were washed once in PBS (5 min, room temp) and incubated with Texas-Red- or Cy2-conjugated secondary antibodies (diluted in 1% BSA in PBS) for 30 min at 37°C in a humid chamber. Cover slips were then washed twice with PBS and mounted onto microscope slides with glycerol gelatin (Sigma-Aldrich, St. Louis) containing 0.1M N-propyl gallate (Sigma-Aldrich, St. Louis) to prevent photo bleaching and were stored at 4°C in the dark until analyzed by confocal microscopy.

### Confocal Microscopy

For confocal microscopy, a Zeiss LSM410 inverted laser scanning microscope was employed. The microscope was equipped with a krypton/argon mixed-gas laser and was operated by the Microcosm Renaissance 410 (v2.3.4) software package. Images were acquired with a Plan-Apochromat 63x/1.4 oil immersion objective (Carl Zeiss, Thornwood). Additional optical magnification (up to 5-fold) was achieved using the zoom feature of the image acquisition software. For two-color analysis, objects were excited using 488/568 nm laser lines. Green and red emissions were recorded through appropriate filters (515–540 nm band pass filter for Cy2 and 590 nm long pass filter for Texas-Red) and stored in separate (red and green) image channels. At the same time, bright field images (Nomarski optics) were collected and stored in a third (blue) channel. Image quality was enhanced during data acquisition using the Renaissance 410 line average feature (8 or 16x).

## Competing Interests

None declared.

## Authors' Contributions

S. K. carried out immunoblot analyses, infectivity assays, and was involved in the construction of plasmids and the production of antibodies. E.M., M.A.K., H.T., S.O., and R.G. participated in immunoblot analyses, infectivity studies, sample preparations, data validation, and overall experimental design. K.S. conceived of the study, performed IFA analyses, and coordinated the study. S.K. and K.S. participated in the writing of the manuscript. All authors read and approved the final manuscript.
